# Building a theory of change to guide fatherhood programming to prevent family violence: a comparison of two programs

**DOI:** 10.1186/s12889-025-24883-7

**Published:** 2025-10-22

**Authors:** Anjalee Kohli, Kate Doyle, Jasmine Uysal, Dickens Ojamuge, Emmanuel Karamage, Rebecka Lundgren

**Affiliations:** 1 Georgetown University, Washington D.C, USA; 2 Equimundo: Center for Masculinities and Social Justice, Brussels, Belgium; 3 Center on Gender Equity and Health, University of California San Diego, 9500 Gilman Drive, Mail Code 0507 La Jolla, San Diego, CA 92093 USA; 4 Save the Children International, 9500 Gilman Drive, Mail Code 0507, Kampala, Uganda; 5 Rwanda Men’s Resource Center, Kigali, Rwanda

**Keywords:** Violence prevention, Violence against women, Violence against children, Interventions, Male engagement, Theory, Domestic violence, Fathers, Parents

## Abstract

**Background:**

Fatherhood programs have increasingly been recognized as a promising approach to prevent violence against women and children (VAWC), yet there remains limited synthesis across programs to clarify the core components that drive change. Most existing studies focus on individual program evaluations. Identifying shared principles or pathways can inform broader implementation and scale. To address this gap, this analysis identified program elements that effectively engage fathers to reduce VAWC by conducting a case analysis of two evidence-based fatherhood programs, Bandebereho and REAL (Responsible, Engaged, and Loving) Fathers, and presenting a joint Theory of Change (TOC).

**Methods:**

Programs were identified through a rapid review of evidence on fatherhood violence prevention programs and selected for their engagement of fathers, published evaluations with positive effects in reducing violence against women and children, adaptation and scale-up in a low resource setting, and having at least one available representative. Authors met regularly over 18-months conducting a desk review of program documentation, building understanding on program strategies and evidence, discussing similarities and differences between the programs, and identifying key elements. Analysis was collated in an excel matrix and utilized to create a pictorial TOC which was reviewed and validated by researchers and implementers from each program.

**Results:**

The TOC highlights program principles, key stakeholders, training, program components, change catalysts, and intermediate outcomes on the pathway to impact. Despite being developed in unique contexts by different teams, programs displayed strikingly similar components and pathways of change leading to measured outcomes demonstrating positive impacts on prevention of violence against women and children.

**Conclusions:**

This TOC reflects evidence-based insights into the core components and pathways through which fatherhood programs may reduce violence against women and children. Although developed from two rigorously evaluated programs, the shared elements identified can serve as a foundation for future research and program development. This initial framework can help guide intervention design, implementation, monitoring and evaluation to promote effectiveness and sustainability in future violence prevention initiatives that engage fathers. Further testing and validation across diverse contexts are needed to assess the theories broader relevance.

## Introduction

Violence against women and children (VAWC) are human rights violations and public health issues of global concern. The two most common forms of violence experienced by women and children occur within the home: intimate partner violence (IPV) and violent discipline by parents or caregivers [1, 2]. Both types of violence have severe negative impacts on the physical and mental health of women and children, as well as on child development [3]. IPV and violent discipline intersect in multiple ways – they frequently co-occur, share common risk factors, are rooted in similar social norms, and lead to similar and compounding consequences over the life course [3]. There is a growing call for integrated and evidence-based programs to prevent VAWC, particularly through gender-transformative social behavior change (SBC) approaches [4, 5]. While still limited, there is emerging evidence that some parenting programs and initiatives that engage men and women using gender transformative strategies can prevent both types of violence, even if not originally designed to do so [5–7]. Gender transformative programs are designed to challenge inequitable gender norms and power imbalances and address the root causes of inequities. They involve both women and men in the process [5, 7].

Parenting SBC programs are typically designed to address violent discipline but not IPV, despite children’s exposure to IPV having similar consequences for children’s health and development, even if not directly witnessed. Few parenting programs focus specifically on engaging fathers or male caregivers [6]. Parenting programs have often either excluded or ignored fathers, involved them only as gatekeepers or supporters of female partners, or struggled to recruit and retrain male participants [5, 6]. Recently, however, several innovative programs have started to actively engage fathers as caregivers, tailoring behavior change strategies and outcomes specifically for men. Some of these programs intentionally focus on fathers and fatherhood, directly recruiting fathers - whether alone or with their partners - and incorporating content that appeals to men’s desires to support their children’s care and development [5].

Some male engagement programs focus on the transition to fatherhood, a life stage when men may be more open to shifting attitudes and behaviors related to discipline, communication, and violence, and to learning new skills to prevent violence against their partners and children [5]. These programs recognize that as men take on roles as caregivers and partners, they must navigate their own abilities, desires, social and gender norms, and the models provided by their own fathers and men in their community [9]. As men step into these new roles and relationships with their partners and their communities, opportunities for learning and growth arise, including space for dialogue and skill-building as they adapt to significant changes in their daily lives. Despite growing interest, evidence on gender transformative fatherhood programs that have demonstrated reductions in both IPV and violent discipline, particularly those that have been scaled to make population level-impact in low-resource settings, is still nascent [10].

Given the emerging state of evidence on scalable gender-transformative fatherhood programs to prevent VAWC, there has been limited synthesis to identify common program components, strategies, and pathways that drive change. Without a grounded, shared understanding not only of which programs work, but why they work, efforts to replicate and scale-up effective fatherhood violence prevention models are significantly constrained. A clearer understanding of shared elements across evidence-based programs, particularly those which have shown scalability, is needed to inform the design and adaptation of future interventions in diverse contexts.

This paper provides a case analysis of two gender-transformative SBC programs designed specifically to engage men as fathers – Responsible, Engaged, and Loving (REAL) Fathers and Bandebereho (“role model” in Kinyarwanda) – which have demonstrated reductions in IPV and violent discipline and are currently being scaled in low-resource settings. Our aim in this analysis is to strengthen understanding on key components and strategies that may be important to engaging fathers to reduce violence in the home. To do this, we [1] describe the REAL Fathers and Bandebereho programs, evaluations, and results [2], analyze the program design, core elements, implementation, evidence, and mechanisms of change, identifying both similarities and differences, and [3], based on this analysis, propose a joint Theory of Change (TOC) for gender transformative fatherhood programming to prevent IPV and violent discipline in the home.

## Methods

We began by identifying gender-transformative fatherhood programs with rigorous evidence supporting their effectiveness in preventing IPV and violent discipline. To identify eligible programs, we conducted a rapid, informal review of the literature, including targeted searches of common academic databases and organizational websites, and drew on prior knowledge of existing gender transformative fatherhood programs. This scan aimed to identify programs with demonstrated impacts on both IPV and violent discipline, particularly those that had progressed beyond the pilot phase and undergone adaptation for scale-up in low-resource settings. Eligibility criteria to include programs in this case analysis included the following: [1] male engagement program with a specific focus on fathers and/or fatherhood; [2] published experimental or quasi-experimental evaluations in peer-reviewed literature; [3] these evaluations must have demonstrated positive effects in sustained (i.e., some period of time post-program) reductions in IPV against women and violent discipline against children; [4] adapted and scaled in low resource settings; and [5] at least one research representative available to participate in the analysis process. We were interested in programs that had been scaled, which describes a process of expanding a program to reach broader populations or new geographic areas while maintaining core components and effectiveness, because these efforts involve adaptation including a detailed review of program strategies, evidence and local consultation to identify essential elements and values to retain at scale. REAL Fathers and Bandebereho, described in detail later in this section, were the only programs identified that met all eligibility criteria. While other fatherhood programs were identified, they were either still in the pilot phase, had evidence of effectiveness for only one type of violence, did not have a representative available to participate, or had not yet been scaled.

### Developing the theory of change

The case-analysis team comprised four core members—two study principal investigators deeply involved in the original design and implementation of REAL Fathers and Bandebereho, a co-investigator, and a project manager/doctoral candidate supporting REAL Fathers’ scale-up—based in the United States and Belgium. Over 18 months, we convened regularly to define: (1) parameters of the analysis; (2) conduct an in-depth desk review of program (e.g., implementation guides, quarterly and learning reports, scale strategy, slide decks) and research documents (e.g., quantitative evaluations, qualitative studies of change pathways, peer reviewed articles); (3) build understanding of program strategies and evidence; (4) map similarities and differences between the programs; and (5) identify key elements for the TOC. Each team member read and reviewed documents. The team also reviewed and discussed existing TOCs related to gender-transformative approaches (e.g., Passages TOC [11], REAL TOC [12], Global Early Adolescent Study TOC [13]). A matrix was developed to capture program principles, change agents, enabling actors, and change catalysts, allowing for a comparison of the critical components, key differences, and similarities between the two programs. This matrix evolved over time as new elements emerged from the analysis and group discussions. The review highlighted important commonalities as well as unique essential elements across the two programs. Next, the team compiled results into an excel matrix and constructed a TOC. The resulting TOC was reviewed and refined by two additional implementation leads from Uganda (for REAL) and Rwanda (for Bandebereho) whose field-based insights were incorporated into the final TOC presented in the Results.

### Positionality and reflexivity

Recognizing our roles as designers, implementers, and evaluators, and as researchers based in high-income countries outside of the program settings, the case-analysis team took deliberate steps to minimize potential bias. All team members independently reviewed source documents before coming together to compare interpretations. We maintained an audit trail of key analytic decisions and held regular reflexive discussions to surface and challenge underlying assumptions. To further guard against confirmation bias, we incorporated feedback from program implementers who had not participated in the initial analysis, ensuring that our TOC reflects both knowledge from program designers and critical, local implementation perspectives. We also acknowledge that the case-analysis team’s positionality as high-income country researchers may influence how we framed and interpreted program elements; future work should further engage additional local stakeholders in ongoing reflexive dialogue to further ground and test the TOC in local contexts.

### REAL Fathers in Uganda

REAL (Responsible, Engaged, and Loving) Fathers is a culturally tailored intervention aimed at preventing IPV and violent discipline by engaging young fathers (aged 16–25 years) of children aged zero to three years old in a community-mentorship program. The program begins with young fathers nominating older, respected men in their community, with whom they have or can develop trusting relationships, to serve as their mentors. Men’s choice of their nominated mentors is validated by their spouse and community leaders. Mentors receive reflective and skills-based training to unpack and transform their gendered beliefs and behaviors, as well as to familiarize them with the program curriculum. Over the course of seven months, mentors guide the young fathers through monthly one-on-one sessions that include activities, reflective discussions, and skill-building exercises, offering personalized support to help young fathers develop new skills and behaviors that strengthen their relationships with their partners and children. In addition, three to four mentors and their mentees come together in monthly group sessions to reflect, learn, share challenges, and motivate each other to practice and maintain new skills. The program involves the young fathers’ female partners in three individual and group sessions focused on communication, parenting, and family planning. Near the end of the seven-month cycle, mentors facilitate a session with female partners to discuss the men’s behaviors, challenges, and how women can support their male partners in sustaining positive changes. To further reinforce the change in norms and behaviors at the community level, a socio-emotional poster campaign is included, promoting positive expectations of fathers and husbands. The program culminates in a community celebration, where young fathers publicly share their achievements and commit to longer-term behavioral changes. This event, attended by wives, community members, and local leaders, ensures that fathers’ commitments are recognized and supported by the community, allowing them to serve as role models for others.

REAL was developed by Save the Children and the Institute for Reproductive Health in response to requests from communities in Northern Uganda, a region that has experienced long-term conflict and consequent disruption to family and social relationships, for a program to support young fathers who lacked elder guidance on how to transition to their new roles as fathers and husbands. The program was initially developed and tested in the Acholi and Lango sub-regions of Northern Uganda between 2013 and 2015 [14]. Between 2016 and 2018, REAL was scaled within those sub-regions and adapted for Karamoja to expand the reach of the program and to test its applicability in a culturally different sub-region [15, 16]. This expansion involved tailoring the program’s approach, language, and monitoring to suit the new context in Karamoja, which was a more rural, pastoralist setting with lower literacy and education levels. While the components of the program remained the same, adaptations to suit the context were critical. For example, mentor training was lengthened, illiterate mentors were paired with literate “senior mentors”, and group sessions preceded individual mentor sessions to help mentors become more comfortable with the content before they mentored on their own. Although the same content was included, the curriculum guide was simplified with less text and more images. Cultural content that did not resonate, such as a “yellow card” drawn from soccer to signal the need for couples to take a break during a disagreement, was replaced with a card with the image of a broken arrow.

A cluster randomized controlled trial (RCT) was conducted in both Northern Uganda and Karamoja to evaluate the program’s effectiveness on violence outcomes. Randomization occurred at the activity level (i.e., by livelihood program in Northern Uganda and by early childhood development (ECD) center in Karamoja). Approximately 300 eligible fathers were recruited into intervention and control groups in each region, totaling 1,193 participants at baseline. The mixed-methods evaluation included surveys with fathers at baseline, endline (eight months post-baseline), and one year post endline (20-months post-baseline), focusing on attitudes, parenting and couple relationships, gender roles, men’s use of violent discipline on children three or younger, men’s IPV perpetration and family planning-related behaviors. Multivariate models were used to assess change over time in the primary outcomes of physical discipline of children and IPV from baseline to endline and baseline to one year post endline among those allocated to intervention versus control. A proportional odds model was used to assess intervention effect over time [6]. The qualitative component included in-depth interviews with a subset of young fathers and their wives to gain insights into their perspectives on changes over time [17]. Finally, after the evaluation was completed, a participatory theory of change exercise was conducted with participating fathers, wives, mentors, and stakeholders to understand their views on the pathways through which REAL and an impact on their families [12].

Like the pilot [14], the evaluation at scale provided strong evidence for REAL Fathers’ effectiveness in preventing IPV against women and violent discipline against young children after seven months of intervention, with these effects sustained one-year post-intervention. Additionally, the study found improvements in secondary outcomes of communication, parent-child relationships, confidence in parenting and use or intent to use family planning. Participants highlighted several key elements that contributed to these outcomes, including improved empathy and emotional connection, communication, shared household responsibilities, reduced alcohol consumption, respect, and livelihoods. These factors were crucial in engaging fathers in parenting, strengthening couple relationships, and preventing IPV and violent discipline [12]. REAL has since expanded to Rwanda and Senegal, with formative research conducted in India, to adapt the program for that context. The program is currently being scaled in six regions across Uganda through partnerships with government- and community-based organizations.

### Bandebereho in Rwanda

Bandebereho (‘role model’) is a gender transformative program that engages fathers and their partners in promoting maternal, newborn, and child health, caregiving, and healthier couple relations. The program targets expectant and current fathers (aged 21 to 35) of children under five and involves them in small group sessions focused on critical reflection and dialogue. Using a structured curriculum, these weekly sessions provide a safe space for men—and their partners, who are invited to some sessions—to discuss and critically examine gender norms, build skills, and practice applying new equitable, non-violent attitudes and behaviors in their daily lives. Inspired by Program P [18], Bandebereho was adapted to the Rwandan context in 2013 by Equimundo: Center for Masculinities and Social Justice (formerly Promundo-US) and the Rwanda Men’s Resource Center (RWAMREC), in collaboration with the Rwanda Biomedical Center, based on formative research and pre-testing with couples in four communities. The resulting program’s 15-session curriculum incorporates participatory activities and group discussion to address issues of gender and power, fatherhood, men’s involvement in reproductive and maternal health, couple communication and decision-making, IPV, caregiving, and child development. RWAMREC piloted Bandebereho in four districts of Rwanda between 2014 and 2015, training local fathers to facilitate the weekly sessions in their communities, with groups of up to 12 couples. Fathers were invited to all 15 sessions; women were invited to participate in eight.

The Bandebereho pilot was evaluated through a two-arm, multisite RCT with 1,199 couples from local communities in Karongi, Musanze, Nyaruguru and Rwamagana districts of Rwanda. Following the baseline in February/March 2015, men and their partners were randomized individually into either the treatment group (*n* = 575 couples) or a control group (*n* = 624 couples). Men were interviewed at four time points: baseline, nine months, 21 months, and six years post baseline. Women were interviewed at nine-month, 21-month, and six-year follow-ups, but not at baseline. The survey assessed topics, including maternal-health seeking, family planning, couple relations, household decision-making, the distribution of childcare and household tasks, responsive care of children, and the use of harsh and physical discipline. While men were asked about IPV perpetration at baseline, only women were asked about IPV at follow-ups. Men were unaware that the women’s questionnaire included questions on violence. The study methods are published in detail elsewhere [19, 20].

The study found that women in the Bandebereho program reported significantly lower rates of physical, sexual, emotional, and economic IPV compared to the control group, with these reductions sustained after six years. Structural equation modeling was used to identify mediators and pathways of change to explain the reductions in physical and sexual IPV. Authors found that no single component was driving reductions in IPV; rather, multiple factors, including improved relationship quality, more gender equitable attitudes, and reduced alcohol abuse among men, contributed to the strong intervention effects [21]. Results also demonstrated that both mothers and fathers who participated in Bandebereho were less likely to physically punish their children than those in the control group, with these effects observed at both 21 months and six years. Additionally, the study found long-term improvements in multiple gender and health-related outcomes, including antenatal care seeking, household decision-making, and parenting practices. Detailed results are published elsewhere [19, 20] and described in the TOC below.

Since 2019, Bandebereho is being scaled through the Rwandan health system, training community health workers to implement the curriculum as part of their routine work. The scaling is overseen by a multi-sectoral advisory group, bringing together Government ministries and institutions in charge of health, ECD, and gender equality. To date, scaling is ongoing in three (out of 30) districts, with nearly 1,500 community health workers trained and more than 50,000 parents reached by the program. Bandebereho was piloted in communities in all four Rwandan provinces, thus no major changes to the program content or approach were required in new districts. However, the curriculum was expanded to 17 sessions to pay greater attention to maternal and child nutrition, in response to current health priorities, and to deepen the focus on men’s shared responsibility for unpaid care work.

## Results

**Fig. 1:**
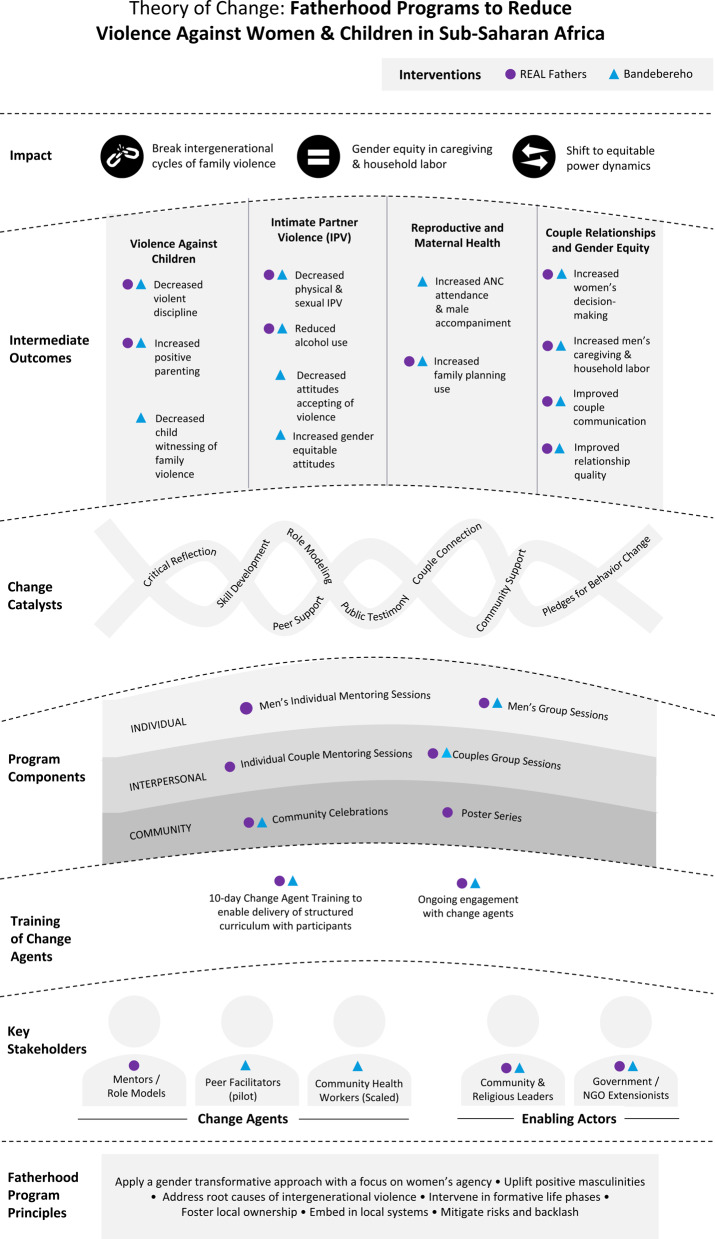
Theory of change for gender transformative fatherhood interventions to reduce violence against women and children in Sub-Saharan Africa

We identified multiple core elements and shared program characteristics important to each program’s effectiveness at preventing VAWC. The resulting TOC (Fig. 1) consists of seven core elements that may underpin positive change in sustained violence prevention: program principles; change agents and enabling actors; training of change agents; program components; change catalysts; intermediate outcomes; and impact (see Table 1 for definitions of these terms). The principles shape the programs, including the program content, approach, and how change agents and enabling actors are engaged in the program. Individual and interpersonal components are designed to activate change catalysts by fostering individual, peer, couple and social investment in new behaviors. Community components support sustained outcomes by making community expectations visible and encouraging individuals to publicly commit to them. In this section, each core element is described, accompanied by examples from each program. Where an element is only relevant to one program, that program is described. The process represented in this TOC unfolds within complex systems, interacting with the physical, political, social, and economic contexts that shape participants’ relationships, lived realities and how they engage with the program. For example, factors such as drought, disease outbreaks, civil unrest, or unemployment may increase stress or financial strain on participating families, but also hinder or disrupt their participation in program activities (e.g., if men migrate to seek work).

**Table 1:** Core elements and definitions for the theory of change underpinning gender transformative fatherhood programs

TOC element	Description
*Program principles*	The values and standards that guide the program’s theory, development, implementation and evaluation to ensure consistency and alignment with program goals and objectives, adherence to ethical considerations, and maintain integrity and effectiveness of the program over time.
*Key stakeholders*	Two key stakeholders are presented – change agents and enabling actors. Change agents are the implementers and facilitators of the program with participants and drive individual and community-level SBC. In addition to program delivery, they may be involved in supporting adaptation, developing community ownership, and scale. Enabling actors are community members and representatives who support program implementation, advocate for, or provide services to the community. Their collaboration and partnership help introduce new ideas and signal leadership support programs.
*Training of change agents*	The process, duration, and content of training provided to the change agents. Training aims to equip change agents with the knowledge, skills, and confidence to deliver the fatherhood program curriculum. This may also include refresher training or support during program implementation.
*Program components*	The activities used to deliver the program’s curriculum and main messages (e.g., knowledge, skills, support) to participants. These components may engage with participants and their families or communities.
*Change catalysts*	The stimuli by which the program components initiate or accelerate shifts in individual and family attitude and behavior changes among intervention participants. These stimuli can be internal (e.g. critical reflection) or external (e.g., public testimony).
*Intermediate outcomes*	Proximal outcomes on the pathway of change to distal impacts. These are the attitude and behavior changes measured through program evaluations.
*Impact*	Distal outcomes and transformation sought by the gender transformative fatherhood programs.

### A theory of change of gender transformative fatherhood programming to prevent IPV and violence against children

#### Fatherhood program principles

The development of both Bandebereho and REAL Fathers was guided by a set of program principles related to fatherhood, with additional principles emerging as the programs were developed, tested, and refined. Programs were adapted to the local context including social and gender norms, policies, and barriers to men’s engagement by involving men, women, community members, and key influencers in program design, implementation, monitoring and evaluation. Both programs were continuously refined based on new evidence and learning throughout implementation and scaling. Although REAL Fathers and Bandebereho were developed independently, they share the following program principles.

- Apply a gender-transformative approach with a focus on women’s agency**.** Both programs go beyond simply providing information regarding gender to men. Instead, REAL Fathers and Bandebereho actively engage men in reflecting on, questioning, and challenging inequitable gender norms and power imbalances between men and women with the ultimate goal to change men’s attitudes and behaviour and promote gender equality. Both programs were informed by women’s preferences regarding men’s roles as fathers and partners, included women in program design and monitoring, and promoted gender equality, power-sharing, and equitable couple relations, including men’s respect for women’s decisions and autonomy in program activities.
- Intervene in formative life phases**.** Both programs assume that fatherhood is a critical period of transition in which men are eager for support and often open to adopting new, positive behaviors. Therefore, they use men’s transition to fatherhood as an entry point to support men to adopt more equitable attitudes and behavior – to be involved, caring, and non-violent fathers and supportive partners, and to raise sons and daughters equally.
- Uplift positive masculinities. Both programs recognize there is no single, fixed definition of masculinity, which describes normative expectations of manhood within a society. Instead, these programs apply an appreciative, diverse approach, acknowledging men’s capacities as caring, non-violent, engaged, and supportive male partners and fathers. They work with men and their communities to promote the adoption of positive masculinities and to be inclusive of a diversity of men.
- Address root causes of intergenerational VAWC. Both programs work with men to prevent violence against their partners and their children by changing attitudes about gender and violence and building knowledge, skills, and coping strategies on how to prevent violence. By preventing children’s exposure to violence (witnessing IPV or indirect exposure), which increases their likelihood of experiencing (women) or perpetrating (men) IPV as adults or using violence against their own children, these programs seek to interrupt intergenerational cycles of violence.
- Mitigate risks and backlash. Both programs acknowledge that promoting men’s greater engagement in their children’s lives and adopting new behaviors aimed at promoting gender equity runs a potential risk of backlash (i.e., denying or minimizing a problem, aggressive opposition, and/or the denial of social and economic assets, opportunities, or inclusion) [6] and take clear steps to prevent and mitigate it. For example, both programs have a strong focus in the curricula on building and promoting positive behaviors and norms and engaging influential social groups to support behavior and norm change, instead of emphasizing negative behaviors, to reduce the risk of backlash [22]. Both programs are willing to support survivors of violence through referral to local services.
- Foster local ownership. Both programs worked with local communities to develop the approach and continue to forge close partnerships with local leaders and government agencies to ensure sustained local ownership and buy-in. This includes establishing local advisory committees or working groups to guide adaptation, implementation, and planning for scale.
- Embed in local systems. Both programs are being scaled through existing government and community systems, such as ECD centers or community health systems, by incorporating training, supervision, funding, recruitment, and implementation into these existing structures.

#### Key stakeholders

Both programs involve key stakeholders at multiple levels to help deliver the program within the community and integrate the program into standard practice at the community and government levels. Though many different stakeholders are engaged throughout the change process in both programs, we present two types of stakeholders below that are critical to program delivery and sustainability.

#### Change agents

Bandebereho and REAL Fathers are delivered to fathers by change agents from local communities who are trained and supported to facilitate their respective gender transformative curricula. Their involvement differs substantially between programs, as Bandebereho takes a group-based approach while REAL Fathers relies on individual mentorship in addition to a group-based approach.

##### Peer facilitators (Bandebereho)

 Change agents in the Bandebereho program are called peer facilitators – community members who have no previous experience facilitating gender transformative approaches. In the pilot, local fathers were trained to facilitate the participatory group sessions of critical reflection, discussion, and skill-building. These fathers participated in the pre-testing of curriculum with their own partners prior to being selected and trained as facilitators. These men met the same criteria as program participants – including being expectant or current fathers of a child under five years, but also had to be literate and have an interest in supporting other men in their community. When scaling through the health system, volunteer community health workers, who are trusted individuals (male or female) elected by their communities, are trained to facilitate the group sessions. Community health workers selected by RWAMREC and the district health authorities to facilitate Bandebereho must be: literate and have completed primary school, ages 25 to 50 years old, motivated to be involved and have time available to implement Bandebereho. Two community health workers, ideally one male and one female, are paired together to co-facilitate the group sessions. They receive a small financial incentive for each cycle of Bandebereho they implement in their communities.

##### Mentors/role models (REAL)

Change agents in REAL Fathers are called mentors, elder men in the community selected by the fathers to provide guidance, advice, support skill building and transformation. REAL Fathers relied on mentors nominated and vetted by the community, building on the finding from formative research that men highly valued mentorship from their elders, especially in the absence of guidance from their uncles and fathers as a result of the prolonged civil war. Mentors are respected individuals from whom young fathers select to receive mentoring. Proposed mentors are vetted by their wives and community leaders prior to training. Mentors are volunteers, although sometimes provided stipend or bicycles for travel to mentor fathers outside of their villages. They are trained to implement the curricula in individual and group sessions.

#### Enabling actors

Bandebereho and REAL both engage community leaders and local government representatives and extension workers to support program visibility, recruitment, implementation, and scaling. These actors play important roles in supporting the training and/or supporting the work of the change agents.

##### Mentors/role models (REAL)

In Bandebereho, community leaders at village level support change agents to recruit participants and support their retention by providing encouragement and (as needed) reminders. They are also invited to the community celebration at the end of the group sessions, alongside other local government representatives. At scale, community leaders at other sub-district administrative levels (above the village level) are also engaged to obtain buy-in and support for implementation and recruitment. In REAL Fathers, community and religious leaders are invited to support participants by joining inception meetings introducing the program in the community and the community celebration at the end of the program.

##### **Government/NGO extensionists (Bandebereho**,** REAL)** 

In both programs a mix of staff from local community-based organizations, non-governmental organizations (NGOs) and local governments support implementation through planning, training change agents, monitoring and supervision. In the scaling of Bandebereho, district authorities in charge of health, gender, and ECD and health system staff at district and sub-district level are key actors supporting program implementation, monitoring, and supervision. The scaling of REAL Fathers is driven by partnerships between community-based organizations and government staff at the district level, with support from national governments. REAL at scale includes integration in ECD and close engagement of district and regional authorities in planning, training, monitoring and supervision.

#### Training of change agents

Training is critical for building change agents’ capacity to facilitate these gender transformative fatherhood programs. Bandebereho and REAL recognize that change agents – the peer facilitators and mentors who deliver the program directly with fathers – need to be supported to reflect on their own gendered attitudes and on men’s and women’s engagement as parents and partners, before being able to support others to do the same. Change agents in both programs receive gender transformative training designed to promote reflection and transformation of their own beliefs, to question inequitable gender norms, and to deliver the program curriculum to participants. This step is crucial to ensuring that facilitators share the beliefs and values of the project and interrogate their own practices and beliefs in relation to gender, violence, and family relationships. For both programs, change agent training is designed to build their familiarity with the curriculum and develop facilitation skills. Training includes opportunities for change agents to practice facilitating the curriculum and receive feedback to build their confidence.

In Bandebereho, the pilot facilitators received an initial ten-day training and a three-day refresher training, after they participated in the pre-testing of the curriculum with their own partners. At scale, different iterations of the training have been tested to identify the best way to equip community health workers to facilitate Bandebereho. Community health workers and their partners first receive a four-day training, a shortened version of the Bandebereho curriculum, to promote couple communication and shared responsibility. Then community health workers receive a ten-day training on how to facilitate the curriculum. The training is split into two five-day trainings implemented over a three-week period, to allow more time for facilitators to absorb and practice the material. In both the pilot and at scale, facilitators receive ongoing supportive supervision, which includes monthly planning meetings and session observation and support (remote and in-person). In the pilot, training was led by RWAMREC; at scale, responsibility for leading the training is being transferred to Community Health Worker Supervisors. At scale, enabling actors, such as the district health, gender and ECD staff and health facility managers and community health worker supervisors who support program implementation and/or provide supportive supervision also receive between four and ten days of training.

Similarly, in the pilot, REAL included a ten-day training of mentors with supportive supervision during implementation. Supportive supervision involved program staff from the implementing organization visiting mentors periodically to observe group sessions, share feedback, and help problem-solve. Mentors and their supervisors were in regular contact throughout implementation to help them prepare for sessions and address challenges. At scale, the approach is adapted to meet the needs of lower literacy mentors. The training is held in two sessions of five days each. One session is provided at the beginning of the project and the other at the halfway point. This adaptation allows lower literacy mentors to facilitate sessions without having to absorb the full curriculum in one sitting. In addition, where lower literacy mentors are engaged, a support structure exists to pair higher and lower literacy mentors in groups of 3–4 mentors for group sessions. These groups work together to prepare for upcoming individual and group sessions, so that lower literacy mentors are supported in reviewing the curriculum.

#### Program components

Both Bandebereho and REAL adopt a structured curriculum which change agents use to guide participants through the change process, which includes activities to promote critical reflection, discussion, and skills-building. Both programs are grounded in participatory approaches to support men (and women) in emotional and relational reflection, discussion, and skill-building related to gender roles, gender equity, parenting and partnership. This is accomplished through activities (e.g., interactive activities, role play, critical reflection, problem-solving, homework) to reinforce gender transformative messaging to promote men’s engagement as equitable, involved and supportive fathers and partners, and to celebrate the changes promoted within the sessions.

Both Bandebereho and REAL are designed to appeal to men’s experiences and desires as fathers and partners. Both programs developed or contextually adapted their curricula through formative research with men and women, collaborative partnerships, and community engagement (e.g., in research, pre-testing and revising content, and providing continuous feedback to strengthen the approach), based on their underlying principles. This allowed each program to tailor the content to respond to men’s lived realities and to build on existing positive norms about fatherhood, while emphasizing a more expansive role for fathers as engaged caregivers and equitable and supportive partners. Change agents emphasize program benefits for men and their families who participate. For Bandebereho, when recruiting fathers to the program, facilitators in the pilot and at scale highlight how the program will help promote their children’s health and well-being and support harmonious family relations. For REAL, men are recruited into the program through community meetings, advice of local leaders, and links with local programming with parents (e.g., ECD). Men propose their mentors; their wives and community leaders vet proposed mentors before recruiting them into the program.

Each component is described below, organized by the level at which the process promotes change, with notations on any differences between programs. The specific activities and program curricula content employed by each program are further specified in Table 2.

**Table 2:** Summary of activities and components across REAL and Bandebereho programs

	REAL	Bandebereho
**Program Overview**		
	**Pilot and Scale**: Program duration: 10 months Curriculum organized around 7 themes (one per month) where each month includes an individual or individual couple mentoring session, a men’s group reflection session or a couple’s group reflection session. Themes are flexible to be adjusted to meet program needs, e.g., including content on immunization or other priority topics.	Pilot: Program duration: Each cycle of Bandebereho takes three to four months. Curriculum includes 15 weekly sessions (men’s partners are invited to 8 sessions). The program originally included 6 couples’ sessions, which were later increased to 8 based on participant feedback. Scale: Program duration: Each cycle of Bandebereho takes four to five months. Curriculum includes 17 weekly sessions (men’s partners are invited to 10 sessions).
Individual (i.e., seeking change through working with men)		
Men’s individual mentoring sessions	**Pilot and Scale**: • 4 sessions • Topics include: fatherhood, tips and tricks for being a REAL father, family dreams and alcohol, loving my family	Not applicable
Men’s group reflection sessions	**Pilot and Scale**: • 3–4 mentors bring their mentees (between 9–16 men) together in reflection and dialogue • 4 sessions • Topics include: fatherhood, tips and tricks for being a REAL Father, family dreams and alcohol, loving my family	**Pilot**: • One facilitator brings a group of 12 men together in discussion • 7 sessions **Scale**: • 7 sessions • 2 facilitators bring groups of 12 men together in discussion • Topics include: fatherhood, caring for a baby, identifying violence, breaking the cycle of violence, alcohol and drug abuse
Interpersonal (i.e., seeking change through working with men and women as couples)		
Individual Couple mentoring sessions	**Pilot and Scale**: • 3 sessions • Topics include: communication, family planning, parenting	Not applicable
Couple group sessions	**Pilot and Scale:**• 3-4 mentors bring their mentees (between 9-16) and their wives together in reflection and dialogue• 3 sessions• Topics include: communication, family planning, parenting	• **Pilot**: **(**8 sessions)** and Scale **(10 sessions)**:** • Maximum of 12 couples in group sessions • Guided discussion, participatory activities such as role play to support couples to practice relationship skills (e.g., communication, conflict resolution, household budgeting) and identify couples’ shared goals for raising children Topics include: gender and sex, men’s participation in antenatal care, childbirth and family planning, raising children, sharing household responsibilities, and making a family budget
Community (i.e., seeking change through engaging women, family and community to celebrate and support men’s behavior change)		
Women’s only group sessions	**Pilot and Scale:**• One women’s only session held to reflect on men’s successes and challenges and women’s experience with REAL Topics include: General feedback on the mentoring project, negative effects of alcohol, gender roles in the home and support between partners, communicating without violence, positive nonviolent child discipline	Not applicable
Poster series	**Pilot and Scale:**• 7 posters, each covering 1 mentoring topic. New posters are displayed each month according to the mentoring topic. (e.g., My husband is a REAL Father – he respects me and I respect him”)• Posters are placed in centrally located public settings to serve as a reminder to men & community that the program is valuable and men are committed to living by these values.	**Pilot: **•, 12 posters depicted positive images of men as caregivers and supportive partners involved in pregnancy, antenatal care, and family planning (e.g., “As a man I care about our child’s health”) • Posters co-designed with the Rwanda Biomedical Center and displayed in health centers and community spaces **Scale: **These were not retained.
Community	**Pilot and Scale:**• One session held after the completion of activities• Involves men, family, community and community leaders • Includes testimony, celebration of achievements and commitments to support and sustain change	**Pilot and Scale:**• Closing ceremony occurs after the completion of the group sessions• Involves men, family, community and community leaders • Includes testimony, celebration of achievements and commitments to support and sustain change

### Individual level (i.e., seeking change through working with men)

#### Men’s individual mentoring sessions

These involve men meeting one-to-one with change agents and participating in learning, experience sharing, self-reflection and dialogue, and skill building. Each mentoring session is structured around a theme. At the end of the session, change agents gave fathers homework and ask for father’s commitment to practice skills. These individual sessions allow men a safe space to share their perspectives and feelings, build trust, and seek advice. Men’s individual mentoring sessions were only applicable to the REAL Fathers program.

#### Men’s group reflection sessions

These sessions create space for men to participate with other men in facilitated reflection and dialogue, share experiences, challenges, motivation and learning, seek advice and encourage each other as they learn new skills and behaviors. Through working in groups, men develop a peer network on their journey to behavior change, building a common identity and support for each other when social pressures may discourage uptake of new behaviors. Both programs used group reflection sessions but had a different number of sessions and session leaders (mentors vs. trained facilitators). Content in both programs was focused on fatherhood, being a good father and influence for the family, including prevention of violence and limiting alcohol intake. Bandebereho had a greater emphasis on caring for infants, breaking the cycle of violence, and drug abuse (in addition to alcohol).

### Interpersonal level (i.e., seeking change through working with men and women as couples)

#### Individual couple mentoring sessions

Change agents meet with couples in their home to engage in couples-based activities, self-reflection, learning, dialogue and skill building. As with the individual sessions, change agents assign homework at the end of the session and seek the couple’s commitment to practice their new skills. These sessions engage the couple as the topics covered are oriented to partners or parents and the curriculum encourages dialogue between the couple on these topics. Individual couple mentoring sessions were only applied in the REAL Fathers program.

#### Couple group sessions

Fathers and their female partners participate in facilitated group sessions with other couples. These sessions are designed to encourage self and couple reflection and dialogue, build skills and gain commitments to practice skills after each session. Couple group sessions allow men and women a safe space to share their perspectives and feelings, build trust, and seek advice. Couple group sessions were applied in both programs, but differed in the number of sessions, whom they were led by, and how structured they were (discussion/reflection vs. structured discussion and activities). Both programs content was focused on communication, parenting, and family planning but Bandebereho included additional content on men’s involvement in antenatal care, sharing household responsibilities, and making a family budget.

### Community-level: (i.e., seeking change through engaging women, family and community to celebrate and support men’s behaviors)

#### Women’s only group session

Change agents meet with women in a facilitated group session with other wives of men engaged in the program. These sessions are designed to engage wives individually to strengthen their awareness and skills to support their husband in building and maintaining parenting skills acquired from the male mentorship sessions. Sessions include discussion and reflection activities on the prior sessions which involved wives and their husbands. Women’s only group sessions were only applied in the REAL Fathers program.

#### Community celebrations

Meetings of men, their partners, change agents and community members that take place at the end of the program. This is a time for men (and women) to share what they have learned, how they have changed, what they have appreciated about the program and their commitments to these behaviors and relationships after the project. Women and community members provide testimony on the changes they have observed in men, their experience with the program and provide social and partner support for men’s continued commitment to these new behaviors. Both programs included community celebrations that were similar.

#### Poster series

Large visual posters are developed and displayed in public gathering points. Posters are designed to convey the communities’ values and perspectives of a good father, man, and husband. Messages complement those emphasized in the mentoring sessions. There were some differences in poster campaigns between the two programs – REAL included seven different posters, to align with each of the sessions, that were replaced monthly. In Bandebereho, posters were static and were placed both in public spaces and health facilities.

#### Change catalysts

We define change catalysts as the processes through which we hypothesize the program, delivered by the change agents, contributes to individual and family-level changes. Although Bandebereho and REAL were developed independently, all catalysts presented are shared between the two programs to varying degrees. Drawing from practice-based evidence and evaluation, change catalysts facilitate the process of change; some motivate and encourage program participation and the sustained adoption of new attitudes and behavior, such as couple connection and peer support.

##### Critical reflection

Both Bandebereho and REAL utilize critical reflection and discussion, a cornerstone of many gender transformative programs, to support participants to consider and question how gender norms and power imbalances are present in their lives. In REAL, critical reflection is facilitated in the change agent training and the mentoring sessions. For the latter, mentors use activities in the curriculum during individual, couple and group sessions to stimulate experience on the topic and then facilitate dialogue and reflection using prompts (e.g., “Do you agree/disagree that children learn best from being hit? Share your views”) in the curriculum. Through this, fathers are asked to explore different ways of being, their attitudes, their family members’ preferences and experiences, and their desires for their family life. Similarly, critical reflection is facilitated in both the change agent training and in the men’s and couples’ group sessions of Bandebereho through participatory activities and structured group discussion. A foundational session in the Bandebereho curriculum supports participants to understand the difference between gender and sex and encourages them to reflect on how gender norms shape their relationships (e.g., “How do these different expectations of how women and men should act influence our roles as parents and how we interact with our children?”) In both programs, critical reflection opens space for men (and women) to see their world differently and uplift protective norms that support parenting and partnership.

##### Skill development 

Both programs support participants to learn new skills in communication, problem solving, child discipline, gender roles, and goal-setting through activities, self and group reflection, and facilitated dialogue. In REAL, mentors facilitate participatory activities and critical reflection with fathers. They reflect on what they learned, their current behaviors, new behaviors, and learn skills to practice these behaviors. Men practice skills they define such as talking to friends or playing with their children to manage stress and build connection. Through demonstration, role play, feedback, homework and support, fathers learn to practice new behaviors. Throughout the month, fathers can return to their mentor, their wives or other REAL fathers for support and guidance as they practice this new skill. As the project focuses on developing new positive behaviors, mentors take an encouraging approach to support fathers. In Bandebereho, information sharing, participatory activities, skills demonstrations and practice, and homework assignments are utilized to build fathers’ skills to care for their children and couples’ skills related to communication, budgeting, and positive parenting. For example, in one session fathers, who are traditionally discouraged from caring for newborns and babies, learn how to properly hold, bathe, and change a baby and practice these skills using locally-made dolls.

##### Peer support

In both programs, group sessions enable men to build relationships with each other through participation in the same program, facilitated dialogue, activities, and reflection that result in friendship and a shared identity as a member of the project. These relationships build a supportive network where men can seek advice from each other and encourage and support each other even when social or peer pressures encourage them to continue their old behaviors. In REAL, men remarked on how having other fathers go through the program supported their own change, especially when pressured by non-participating peers to deviate from their new behaviors. Similarly, Bandebereho participants highlighted the importance of relationships built among couples in the group to support behavior change and to update them on the curriculum in case they had to miss a session. Activities and group discussion throughout the curriculum encourage participants to identify ways that they can support each other in making positive changes in their relationships. The relationships and peer support developed in the group often continues even after completing the curriculum. Some groups continue to meet and some have even established their own savings and lending groups to support men and couples in achieving their goals for their family.

##### Role modeling

Both programs utilize the concept of role models, respected individuals whose behavior is held up as an example for others to follow/learn from, to support behavior change. In REAL, mentors can be role models, using their lived experience to show men new ways of being in partnership and being fathers. In addition, through men’s participation in REAL’s group sessions, men learn from other fathers and their adoption of new skills and the changes they are making in their family life. These opportunities allow men to engage with peers for support and as role models to inspire their behavior change. REAL fathers are also role models for other fathers and couples in the community who want to realize the benefits REAL families experience. Similarly, in Bandebereho (which translates to ‘role model’ in Kinyarwanda), facilitators and group participants are encouraged to make positive changes, by adopting healthy and more gender-equitable behaviors, that can enable them to become role models for each other, their children, and the community. The program is careful not to publicly label facilitators or participants as role models, to avoid the idea that any one person is perfect. Rather, the program emphasizes that change is a gradual and continuing process and becoming a role model is something to which facilitators and participants can aspire.

##### Couple connection

Both programs aim to support healthier couple relations by building emotional connection and understanding between men and their partners. This connection is supported by building communication skills and creating spaces and opportunities for couples to discuss and listen to each other. This helps to build trust and strengthen couples’ relationships, and allow them to set goals together, particularly in contexts where spaces for couple communication is not normative. This strengthened connection enables partners to support each other in adopting new behaviors and sustaining positive changes. REAL supports couple connection through wives participating in select mentoring sessions. In the sessions, this includes having couples select an issue, discuss possible answers, and come to a decision. Fathers describe how the program deepens their connections with their wives and stimulates conversation on topics such as emotions, parenting, and planning for their family. For Bandebereho, couple connection and communication are fostered through couple sessions, which comprise more than half the sessions, and homework assignments, which encourage partners to discuss, set goals, and make decisions together. For example, an activity in one session supports couples to jointly develop a vision for their child’s future and has them discuss how they can work together to achieve that vision, including any changes they need to make.

##### Community support

Both programs work with the broader community to be champions of the program and support the new behaviors adopted by program participants. Community engagement also signals to the change agents that their role in delivering the program is supported and respected. REAL and Bandebereho gain the support of relevant community leaders by engaging with them early when the project is introduced and assessing their commitment to its objectives. For Bandebereho this also includes a four-day gender transformative training for district-level leaders (e.g., district health, gender, ECD and social affairs officers) to enable critical reflection of their own gendered attitudes and to build their understanding of the program and its aims. In both programs, community leaders were involved in identifying potential program participants, acting as advisory members, and facilitating public testimony, posters and community celebration events. In Bandebereho, support from local leaders has been crucial for recruiting participants and encouraging them to remain involved. Men and women also report that the involvement of local leaders demonstrates their appreciation and value of the program, which motivates participants to sustain the positive changes made.

##### Public testimony

Public testimony provides an opportunity for participants to share the meaningful changes and benefits they have experienced from participating in the program and offers an example to others that change is possible and desirable. Thus, public testimony both serves to reinforce individual behavior change and spread this change through social networks in the community which have not directly benefited from the program. In REAL and Bandebereho, testimony occurs both within the group sessions and in public spaces through the community celebrations. In the group sessions, fathers (and their partners) share the changes they are making within the facilitated activities and group discussion. During the community celebrations at the end of each cycle of REAL, men (and their partners) publicly state the changes they have made and their commitment to sustaining these changes going forward. In Bandebereho, the public testimony aspect of the program developed organically, as participating men and women were eager to share the positive changes they had made and to encourage others to adopt new behaviors. Over time, this developed into the more structured opportunity to provide testimony at the closing ceremonies. Occasionally, participants are also invited to give public testimony at monthly community gatherings organized by local leaders on issues affecting families in the community.

##### Pledges for behavior change

 In both programs, men publicly pledge to learn and adopt the new behaviors they learned in front of their partners, other men, and community members. These public pledges create a social obligation for men to live by their commitments and also allow for social accountability, where other witnesses, including female partners, may follow up with men to encourage and correct their behaviors. The final session of Bandebereho asks couples to reflect on the positive changes they have made and to create an action plan to sustain the changes and work towards achieving their family vision. These action plans are voluntarily shared within the group and often include pledges for further behavior change. In REAL, public pledges are part of a community celebration, honoring men and their partners for their efforts and commitment to healthy families.

### Outcomes

REAL and Bandebereho sought and achieved impact on similar outcomes, as measured in their impact evaluations. In this section, we highlight the evidence related to the four domains of intermediate outcomes highlighted in the TOC: IPV, violence against children, reproductive and maternal health; and couple relationships and gender equity. For some outcomes, only one program measured the effect; so, we specify where evidence is available from only one of the two programs.

#### Intimate partner violence

##### Decreased intimate partner violence

Both Bandebereho and REAL demonstrated reductions in IPV. The Bandebereho RCT found that participating women reported significantly lower rates of past-year physical, sexual, emotional, and economic IPV compared to the control group at 21 months [19, 21]. A six-year follow-up demonstrated long-term effects on IPV, including physical (OR 0.45, 95% CI 0.34–0.60 *p* < 0.001), sexual (OR 0.50, 95% CI 0.37–0.67, *p* < 0.001), economic (OR 0.47, 95% CI 0.34–0.64, *p* < 0.001), moderate or several emotional (OR 0.40, 95% CI 0.29–0.56, *p* < 0.001) [20]. The REAL evaluation at scale found that fathers reported greater odds of not using physical and/or sexual IPV compared to frequent IPV use at endline (Karamoja: AOR 3.45, 95% CI 2.15 -5.53; Northern Uganda: AOR 2.15, 95% CI 1.28 - 3.63). One year after intervention, men reported greater odds of not using physical and/or sexual IPV than baseline in Karamoja (AOR 3.20, 95% CI 2.09- 4.90) and Northern Uganda (AOR 2.90, 95% CI 1.51-5.58) [6]. These findings support the pilot evaluation (2015–2017) in Northern Uganda which also found a significant and sustained decline in any IPV and psychological IPV [14].

##### Reduced alcohol use

In Bandebereho and REAL, men reported reduced use of alcohol, a known risk factor for IPV and violent discipline of children. In Bandebereho, participating men reported less harmful alcohol consumption at 9 and 21-months. Long-term effects were seen at six year follow-up, where men reported less harmful weekly alcohol consumption – drinking until getting drunk – compared to the control group (OR 0.25, 95% CI 0.14–0.46, *p* < 0.001), a finding affirmed by men’s female partners (OR 0.47, 95% CI 0.33–0.68, *p* < 0.001) [20]. In REAL, men in Karamoja reported significantly less daily drinking of alcohol at endline (14.5%) compared to the control group (29%). In Northern Uganda, significantly more men reported not consuming alcohol at endline (80.8%) than in the control group (73.0%) [15, 16].

##### Increased gender equitable attitudes

The Bandebereho RCT found that compared to the control group, participating men reported attitudes less accepting of IPV and more equitable attitudes about men’s and women’s roles at nine months [21]; similar effects were seen at 21 months (unpublished).

#### Violence against children

##### Decreased violent discipline against children

Bandebereho and REAL both demonstrated reductions in parents use of violent discipline. The RCT of the Bandebereho pilot found self-reported decreased use of past-month physical punishment of children by both men (AOR 0.66, 95% CI 0.50–0.88, *p* < 0.01) and women (AOR 0.56, 95% CI 0.41–0.79, *p* = 0.001) compared to the control group at 21 months [19]. At six-year follow-up, couples continued to report lower rates of physical punishment compared to the control group, although with smaller effects (men: AOR 0.72, 95% CI 0.57–0.92, *p* = 0.009; women: AOR 0.68, 95% CI 0.49–0.93, *p* = 0.017) [20]. Couples also reported less supportive attitudes towards physical punishment of children. The REAL evaluation found that at endline, fathers reported greater odds of not using physically violent discipline with their young child in both study sites (Northern Uganda: AOR 1.98, 95% CI1.34-2.93; Karamoja: AOR 2.14, 95% CI 1.46- 3.12). One-year after intervention, fathers reported even less use of physically violent discipline with their young children in Northern Uganda (AOR 2.79, 95% CI 1.92 - 4.05) and Karamoja (AOR 3.59, 95% CI 2.53- 5.10). Fathers also reported attitudes rejecting of physical child punishment at endline and one-year after intervention [15, 16].

##### Increased positive parenting 

Positive parenting includes actions or words to encourage behaviors. Men participating in the REAL Fathers scale reported engaging in significantly more positive parenting at endline (Northern Uganda: AOR 1.88, 95% CI 1.21- 2.94; Karamoja: AOR 2.06, 95% CI 1.35- 3.16) and one-year later (Northern Uganda: AOR 1.62; 95% CI 1.06- 2.47; Karamoja: AOR 2.43, 95% CI 1.65- 3.60). Fathers reported increased quality time interactions with their child (e.g., singing, playing, etc.) at endline [15, 16]. Findings from the qualitative evaluation conducted with REAL Fathers and their wives found that parents reported adopting new positive strategies to discipline their children in place of corporal punishment and yelling. They described improved relationships with their children and fathers spending more time with their children [17]. The Bandebereho RCT found improvements in parent-child interactions that support child development. At 21 months, compared to the control group, participating men and women spent more time daily teaching their children (men: 25 vs.18 min; women: 37 vs. 30) and men spent more time telling stories, singing, or playing with children (24 vs. 15 min). Forthcoming analysis from the six-year follow-up of the Bandebereho RCT found that participating men and women reported greater support for positive discipline and use of positive parenting practices [23].

##### Decreased child witnessing of family violence

In addition to the lower rates of IPV reported by women, the Bandebereho RCT found that women in Bandebereho reported that their children were present during an act of IPV less often than those in the control group at 21 months and six years (unpublished). This was not measured in the REAL Father’s evaluation; however, we hypothesize that through reduced IPV experience reported by women, children would similarly have reduced exposure to witnessing IPV.

#### Reproductive and maternal health

##### Increased family planning use

The RCT of the Bandebereho pilot demonstrated increased use of modern contraceptives by men (AOR 1.65, 95% CI 1.24–2.20, *p* = 0.001) and women (AOR 1.53, 95% CI 1.15–2.04, *p* = 0.004) in Bandebereho compared to the control group at 21-months [19]. This finding was not sustained at six-year follow-up. The evaluation of REAL in Northern Uganda showed that fathers reported increased use of modern family planning at endline (AOR 2.3, 95% CI 1.46- 3.64) with findings largely sustained one-year later (AOR 1.66, 95% CI 1.14- 2.42). In Karamoja, results on family planning were not as strong, though at the time of the study, modern family planning methods were not available at many clinics. Fathers did report increased use of any family planning method at endline (AOR 2.83, 95% CI 1.78- 4.56) [15, 16].

##### Increased antenatal care attendance and men’s accompaniment

At 21-month follow-up, couples in the Bandebereho pilot reported greater antenatal care (ANC) attendance by women (IRR 1.09, 95% CI 1.05–1.14, *p* < 0.001) and accompaniment of men to ANC (for women: IRR 1.50, 95% CI 1.36–1.65, *p* < 0.001; for men: IRR 1.33, 95% CI 1.23–1.45, *p* < 0.001) compared to the control group [19]. Long-term effects were found on ANC attendance by women (IRR 1.03, 95% CI 1.00–1.05, *p* = 0.029) and accompaniment by men (IRR 1.29, 95% CI 1.19–1.40, *p* < 0.001) at six-year follow-up [20]. At 21-months, women also reported greater partner support during pregnancy (emotional, spiritual, financial, material, etc.) compared to the control group (outcome not measured at six year follow-up).

#### Couple relationships and gender equity

##### Increased women’s decision-making

The Bandebereho RCT found reduced male dominant household financial decision-making reported by participating women (AOR 0.31, 95% CI 0.24–0.42, *p* < 0.001) and men (AOR 0.35, 95% CI 0.25–0.48, *p* < 0.001) compared to the control group at 21 months; effects were maintained at six-year follow-up. At 21 months, less dominance by men in decisions about the number, timing and spacing of children was reported by women (AOR 0.57, 95% CI 0.45–0.72, *p* < 0.001) and men (AOR 0.48, 95% CI 0.36–0.63, *p* < 0.001) compared to the control group [19], but not at six-year follow-up. Participation in REAL Fathers was associated with significantly increased shared household decision making at endline compared to baseline (Northern Uganda: AOR 1.63, 95% CI 1.13-2.34; Karamoja: AOR 3.56, 95% CI 2.12-6.08); these were sustained one-year later [15, 16].

##### Increased men’s caregiving & household labor

The Bandebereho RCT found that, compared to the control group, participating men and women were more likely to report sharing of childcare and household tasks between partners (for men: beta 0.33, 95% CI 0.26–0.41, *p* < 0.0001; for women: beta 0.39, 95% CI 0.31–0.47, *p* < 0.0001) and greater time spent by men on such tasks (beta 0.86, 95% CI 0.50–1.22, *p* < 0.001) at 21 months [19]. Similar effects were seen at six-year follow-up [20]. REAL Fathers had a positive effect on gender equitable attitudes in caregiving and childcare at endline (Northern Uganda: AOR 2.97, 95% CI: 1.67-5.60; Karamoja: AOR 3.25, 95% CI: 2.21-4.84) [15]; these findings were sustained one-year later [16].

##### Improved couple communication

The Bandebereho RCT found greater frequency of couple communication on a series of household decisions reported by women and men in the program compared to the control group at 9 months as well as satisfaction with couple communication [19]; improved communication was also found at 21 months and six years (unpublished). The evaluation of REAL Fathers at scale demonstrated greater couple communication at endline (Northern Uganda: AOR 2.13, 95% CI: 1.37-3.33; Karamoja: AOR 1.94, 95% CI: 1.32-2.85) and one-year later in Northern Uganda (AOR 1.51, 95% CI: 1.00-2.27) [15].

##### Improved relationship quality

The Bandebereho RCT found that men and women reported greater relationship closeness (a composite examining appreciation, affection, and respect between partners) and less quarreling at nine months compared to the control group [21].

### Intended impact

Through their gender transformative work with fathers and their partners, the Bandebereho and REAL Fathers programs sought to contribute to long-term impacts to support non-violent, equitable, healthy and caring families. The hypothesized program impacts are described below, including the pathways of change.

#### Break intergenerational cycles of family violence

Both REAL and Bandebereho acknowledge the intergenerational transmission of violence and seek to break this harmful cycle. The programs seek to prevent violence not just by raising awareness of violence and its consequences, but by developing skills that support non-violent relationships such as communication, emotional regulation, conflict resolution, and positive parenting. By strengthening parents’ own relationships and skills, the programs aim to support children to be raised free from violence and with models of healthy, non-violent and communicative partnership – promoting the intergenerational transmission of care and equity, rather than violence. We hypothesize that the children of participants and subsequent generations may see reductions in experience and perpetration of violence, based on the existing program effects on IPV, violent discipline, positive parenting, and couple relations.

#### Shift to equitable power dynamics

REAL Fathers and Bandebereho both seek to shift harmful gender norms that underpin inequitable power dynamics between men and women in the household. Studies show that households with male-dominant power structures significantly increase the risk for male-to-female violence while egalitarian household structures have been shown to be protective against violence in the home and associated with increased perceptions of fairness, communication, and marital satisfaction [24]. Additionally, households where women have low decision-making power report higher morbidity for women and higher morbidity and mortality for children [25]. Thus, both programs strive to address norms that underpin inequitable gender power dynamics and work with men and women to promote more equitable relations, including increased participation and power of women in decisions on key issues such as reproductive health, spending of household resources, and childcare and education.

#### Gender equity in caregiving and household labor

REAL and Bandebereho strive to promote an equitable division of unpaid household labor – including caregiving and household tasks – between men and women. Gender norms have traditionally assigned a rigid division of labor within the home, with childcare and household tasks seen as women’s responsibility. Both programs target shifting the gender norms that underpin this gendered division of labor, including gender norms that men should not be involved in caregiving or household chores and that women are naturally better suited for care work [26]. They encourage men (and build their skills and confidence) to share childcare and household tasks with their partners. A more equitable division of household labor and caregiving can increase couple communication and feelings of fairness to promote egalitarian, healthy relationships [27] and increased bonding with children [26], which further supports violence prevention efforts. Both programs saw an improvement in gender equitable attitudes and men’s caregiving via intermediate outcomes, supporting the programs’ impact towards gender equity in caregiving and household labor.

## Discussion

Despite growing interest in parenting and male engagement approaches to tackle VAWC in the home, there is limited information on effective and scalable intervention designs. This is a critical gap, given the importance of understanding processes of change for program adaptation, scale-up and sustainability. This article examines two well-known fatherhood interventions, REAL Fathers and Bandebereho, that address the intersections of violence against women and children and have demonstrated effectiveness and scalability. While successful programs like these pave the way for future efforts, on their own they are unlikely to solve the challenge of implementing violence prevention efforts at a scale that would bring about lasting, widespread reductions in VAWC. To reach that goal, identifying and funding evidence-based programs, in addition to funding innovative new programs and strategies, is essential but not sufficient. It is also imperative to identify the core elements and change mechanisms of effective programs to guide their adaptation in new contexts and to inform the development of new programs suitable to the given dynamic and complex implementation landscape. With this knowledge, violence prevention efforts can nimbly respond to shifting contexts over space and time to expand program impact [28].

This paper presents a Theory of Change (TOC) which transcends each individual program to inform the design, adaptation, and scale of gender transformative programs with fathers to interrupt the intergenerational transfer of violence. We compare and contrast both the similarities and slight differences between the programs, acknowledging that these differences might influence feasibility, adaptability, and ultimately, transferability into new settings. Importantly, both programs were designed based on research that provided nuanced understanding of what is means to be father/man in the context, allowing the program design to build on positive values and norms for a culturally supported change process. This likely contributed to their effectiveness and reduced any backlash or negative consequences. Yet, it is notable that although these two programs were developed by different organizations and in different contexts, they have very similar principles and core components. Both include principles which are commonly seen in gender transformative approaches designed for scale, such as a focus on women’s agency and local ownership, efforts to embed interventions in local systems and mitigation of backlash [29]. These programs use a constellation of change catalysts commonly recognized as essential attributes of social norms shifting programs such as critical reflection, public testimony, role modeling and public pledges and support [30].

This TOC can guide the adaptation of existing SBC programs or design of new ones, as well as their implementation. The results of this examination suggest the core principles and values underlying these interventions may be critical to their success. Unique to these two programs is their attempt to address the root causes of intergenerational violence by engaging men during a formative period in their life. A hallmark of these programs is their focus on positive masculinities, a critical element of success, as it motivates men to participate and do the hard work of change, based on their desire to fulfill emotionally and socially rewarding roles.

The notable similarities between the program principles, change agents, enabling actors, training, intervention components and change catalysts across these two proven programs suggest that these components are drivers of behavioral change and should be considered in future, similar initiatives. However, this TOC is drawn from only two programs based in two countries in East Africa, and it will be important to validate it with the results of other effective approaches, both within the region and globally —including in urban LMIC settings, among adolescent fathers, and in crisis-affected contexts. Similarly, further research to understand change pathways and mechanisms of change would further clarify to how and to what extent the hypothesized change catalysts drive change and for whom. Although some related interventions have measured processes like reflective dialogue or community mobilization or examined how participants internalized learning and changed their behaviors [12], systematic operationalization of these catalysts remains scarce; future studies should therefore co-develop measurement plans with local stakeholders and integrate mixed-methods, participant-driven assessments to capture pathway strength and variation. This is particularly important as change catalysts themselves, may, in some instances hinder program success; for example, mentors or peer role models who retain inequitable gender norms can inadvertently reinforce the status quo. Experts in norm-shifting interventions have repeatedly called for explicit measurement, monitoring, evaluation, and refinement based on arising negative unanticipated consequences [22]. While both REAL and Bandebereho employed adaptive management [31], monitoring for positive or negative shifts and making regular modifications based on this evidence, systematic reporting on negative or unintended backlash remains rare in the peer-reviewed literature. We also acknowledge limitations in the evaluation designs of both programs: potential selection biases from non‐random attrition, information biases arising from reliance on self‐reported measures of sensitive outcomes, unmeasured confounding, and—in one evaluation—the absence of baseline data for the women’s cohort. These factors could influence effect estimates and the robustness of causal inferences, underscoring the need for further rigorous evaluation. Despite our limitations, this TOC can support expansion of fatherhood programs including efforts to institutionalize evidence-based fatherhood programs in health and ECD systems. Though not covered in this paper, efforts are underway to integrate REAL in ECD programming nationally in Uganda and to institutionalize Bandebereho, including program training, implementation, monitoring and supervision, in the Rwandan health system [29, 32].

## Conclusion

The pathway to sustained global reduction of violence by fathers is unlikely to come from replicating any single program, but rather through recognition of the core elements that enable effective gender transformative fatherhood programming to prevent violence. The TOC presented here, grounded in two rigorously evaluated programs, offers a foundation for informing broader programming and education efforts at local, national, and global levels. These insights can support the development and adaptation of strategies to effectively engage fathers to prevent family violence, while remaining responsive to diverse cultural and contextual realities. Further research and testing are needed to validate and refine this theory of change across different settings to guide fatherhood intervention design, implementation, monitoring, and scale-up.

## Data Availability

This article is a comparative case analysis based on a desk review of published studies and program documentation; no new individual-level participant data were collected or analyzed. The minimal materials necessary to interpret and build upon the synthesis are provided as supplementary files: a structured evidence/methods matrix comparing program principles, components, and outcomes and the joint TOC figure. All publicly available sources informing the analysis are cited in the References with persistent links where available. Some program implementation documents consulted for context were provided by Save the Children Uganda/Institute for Reproductive Health (Georgetown University) and the Rwanda Men’s Resource Center (RWAMREC). These third-party materials are not publicly available due to organizational confidentiality restrictions, but may be available from the corresponding author on reasonable request and with permission of the respective organizations.

## Abbreviations

ECD: Early childhood development
IPV: Intimate partner violence
NGO: Non-governmental organization
REAL: Responsible, Engaged and Loving Fathers
RCT: Randomized control trial
RWAMREC: Rwanda Men’s Resource Center
SBC: Social and behavior change
TOC: Theory of change
